# The frequency of using certain feedback methods in the teaching of medicine: a survey of teachers at the medical faculties in Baden-Wurttemberg

**DOI:** 10.3205/zma001253

**Published:** 2019-08-15

**Authors:** Kevin Kunz, Mirka Burkert, Felix Heindl, Katrin Schüttpelz-Brauns, Marianne Giesler

**Affiliations:** 1 Universität Freiburg, Medizinische Fakultät, Kompetenzzentrum Evaluation in der Medizin Baden-Württemberg, Freiburg, Germany; 2 Universität Heidelberg, Medizinische Fakultät Heidelberg, Kompetenzzentrum für Prüfungen in der Medizin Baden-Württemberg, Heidelberg, Germany; 3 Universität Ulm, Medizinische Fakultät, Kompetenzzentrum E-Learning in der Medizin Baden-Württemberg, Ulm, Germany; 4 Universität Heidelberg, Medizinische Fakultät Mannheim, GB Studium und Lehrentwicklung, Mannheim, Germany

**Keywords:** feedback, feedback methods, medical education, formative feedback, competency-based education

## Abstract

**Objectives: **Feedback is one of the most important methods for competency-based teaching. A survey was conducted to learn more about the use of feedback methods at five medical faculties.

**Methods:** In the 2017 summer semester, teachers at Baden-Wurttemberg’s medical schools in Freiburg, Heidelberg, Mannheim, Tuebingen and Ulm were invited to participate in the survey. The link to the questionnaire was sent to the teaching coordinators at the various departments at each of the five medical schools. The teaching coordinators were asked to forward the link to the questionnaire to all instructors in their department. At one location, all instructors were directly addressed. The data were collected online.

**Results: **A total of 464 instructors participated in the survey. Most consider feedback in medical education as important (23%) or very important (72%). However, some feedback methods are hardly used. The reason for this is, in particular, that some of the feedback methods are unfamiliar, e.g. checklists (56%), or not considered necessary by the instructors, e.g. written feedback (31%). Fifty-five percent of the instructors would like to receive further education or information on feedback.

**Conclusion:** The results show that the use of feedback methods in medical teaching is expandable and that teachers find feedback to be important. Accordingly, nothing should stand in the way of a greater use of feedback methods in teaching. However, in order for this to happen, it is important that instructors are made more familiar with feedback methods.

## Zusammenfassung

**Zielsetzung: **Feedback ist eine der wichtigsten Methoden bei der Umsetzung kompetenzorientierter Lehre. Um mehr über den Einsatz von Feedbackmethoden an den Medizinischen Fakultäten in Baden-Württemberg zu erfahren, wurde eine Bestandsaufnahme an fünf Fakultäten durchgeführt.

**Methodik: **Im Sommersemester 2017 wurden Lehrende der Medizinischen Fakultäten in Freiburg, Heidelberg, Mannheim, Tübingen und Ulm zum Thema Feedback befragt. Der Link zum Fragebogen wurde an die Lehrkoordinatorinnen und Lehrkoordinatoren der verschiedenen Fächer an den einzelnen Standorten verschickt, mit der Bitte, diesen an alle Lehrenden in ihrem Fach weiterzuleiten. An einem Standort konnten alle Lehrenden direkt angeschrieben werden. Die Daten wurden online erhoben.

**Ergebnisse: **Insgesamt nahmen 464 Lehrende an der Befragung teil. Die meisten Lehrenden sehen Feedback in der medizinischen Ausbildung als wichtig (23%) bzw. sehr wichtig (72%) an. Einige Feedbackmethoden werden jedoch kaum genutzt. Gründe hierfür sind vor allem, dass einige Methoden nicht bekannt sind – z.B. Checklisten (56%) – oder von den Lehrenden als nicht erforderlich für ihre Lehrveranstaltungen eingestuft werden, z.B. schriftliches Feedback (31%). 55% der Lehrenden wünschen sich Fortbildungen oder Informationen zum Thema Feedback.

**Schlussfolgerung:** Die Ergebnisse zeigen, dass der Einsatz von Feedbackmethoden in der medizinischen Lehre ausbaufähig ist und dass die Dozentinnen und Dozenten Feedback als wichtig empfinden. Demnach sollte einer stärkeren Nutzung von Feedbackmethoden in der Lehre nichts im Weg stehen. Damit dies jedoch umgesetzt werden kann, ist es wichtig, dass den Lehrenden die einzelnen Methoden bekannter gemacht werden.

## Introduction

The development and fostering of competencies is directly connected with the feedback received by students from teachers [1], [2], [3], [4]. Without targeted feedback, good academic performance by students cannot be confirmed or solidified, and mistakes go uncorrected [5]. This makes feedback one of the central methods for implementing competency-based teaching and developing competencies [6], [7]. The aim of feedback is to give students information about their academic progress in terms of defined aspects of medical practice respectively personal competencies and skills in a way that shows potential for further development so that a student’s performance is constantly being improved [5], [8]. Accordingly, giving constructive feedback is viewed by students as the most important activity a teacher can engage in [9]. In addition, teachers who regularly give feedback are rated higher by students [10]. In contrast, studies show that many medical students have the impression that they receive specific feedback too seldom from teachers [11], [12], [13]. This corresponds with the evaluations of the German Council for Science and Humanities (Wissenschaftsrat) which evaluates teacher engagement in medicine as inadequate in terms of giving feedback and urges more intensive mentoring [14]. So that a constructive process for providing feedback and good mentoring of students can be ensured, it is important to train instructors in how to give feedback and familiarize them with the relevant standards [5], [11].

There are currently no publications in the German-speaking countries that shed light on which of the common feedback methods identified in the literature are being used by medical faculties or with what frequency during the course of their teaching. Hence, a survey was taken on the use of feedback methods at Baden-Wurttemberg’s medical schools as part of the BMBF-funded MER*LIN* (Medical Education Research – *Lehrforschung im Netz BW*) project promoting the development of competency-based medical education in Baden-Wurttemberg. The aim of this survey was to identify which feedback methods are currently being used in medical teaching and for which reasons certain methods are not being utilized. The results of this survey are intended to contribute to optimizing the use of feedback methods in teaching medicine.

## Methods

### Survey procedure

The feedback methods that can be used in teaching medicine were identified based on current research. At the Baden-Wurttemberg Competence Centre for Evaluation in Medicine, a total of 35 questions were then developed from existing questionnaires.

The resulting questionnaire contains questions on the importance of feedback, frequency of use for individual methods, and on reasons for not using methods. Most questions were assigned a four-point Likert scale. In addition, there were single-choice questions and the option to provide free-text commentary. More than one response was possible for some of the questions. To gain an overview of the use of feedback methods in the courses, the instructors were asked for information regarding the frequency (1=never to 4=very frequently) with which they apply certain feedback methods (verbal, written, technology-based, checklists and peer feedback) in their teaching. In addition, questions were asked about familiarity with and knowledge of selected feedback methods. To ascertain the level of knowledge instructors possess regarding the individual methods, they were asked to rate their familiarity with common feedback methods in the literature on a four-point scale (1=method unknown; 2=unknown, more information desired; 3=known, not used; 4=known, used). 

For the purpose of this survey, a rough differentiation was made between less formal and less standardized methods (e.g. verbal or written feedback) and standardized feedback methods. Standardized methods follow a specific procedure and use checklists or questionnaires to evaluate student performance. Examples include mini-CEX (Mini Clinical Evaluation Exercise), DOPS (Direct Observation of Procedural Skills) and CEC (Clinical Encounter Cards) [2], [12], [15], [16], [17]. Technology-based feedback (e.g. audience response systems) would fall under the category of standardized methods. Also, there are methods that can be carried out in either an informal or standardized manner, such as peer feedback. Likewise, the survey asked about which form and in which situations teachers give feedback and if there is a desire for information or training on the topic of feedback. 

The questionnaire was first administered in a pilot study conducted during the 2016 summer semester at the medical faculty in Freiburg. Teaching coordinators were surveyed. The questionnaire then underwent revisions that supplemented or reformulated questions; however, the content remained unchanged. After revision, the questionnaire was tried out on a small group of practicing physicians at the University Hospital in Freiburg and on employees in the MER*LIN* project in Freiburg to verify the suitability of the questionnaire and make any further changes.

In the 2017 summer semester, the revised questionnaire was administered online to the medical faculties in Freiburg, Heidelberg, Mannheim, Tuebingen and Ulm. The aim was to survey all instructors in medicine at each campus who taught in the first and second study phases and the final year. The time for the survey was six weeks. After two and four weeks the instructors were each sent a reminder. The instructors were contacted via email by staff members of the MER*LIN* project at each university. At four universities the teaching coordinators for the first and second study phases were sent information by email about the survey. They were requested to forward this information to all of the instructors in their subject area. At one campus, all of the teachers and final-year mentors were contacted directly by email. No statement can be made about the number of forwarded emails or the total number of teachers who received the invitation to take the survey. Hence, it was not possible to calculate a response rate. Overall, counting all campuses around 1,200 teachers were contacted directly by email.

Survey participation took place by following the link which was sent to the instructors in the same email containing the invitation. Data were collected using EvaSys survey software. The instructors were assigned numerical codes to protect their anonymity during analysis and presentation of the results. Participation in the study was voluntary for the instructors.

#### Sample

A total of 464 instructors participated in the survey. Of these, 60% were male. Twenty percent of the instructors had less than five years of teaching experience, 28% had 5-10 years, 33% had 11-20 years and 19% had more than 20 years. In reference to teaching experience, there was no significant difference between the locations (χ^2^=19,84, df=12, p=.07, w=.207). However, gender differences could be determined: overall, male instructors had more experience than female ones (χ^2^=29,08, df=3, p=.00, w=.250). The other analyses show that 36% of the surveyed instructors teach in the first phase of study, 83% in the second phase and 60% in the final practical year. Analysis of survey participation for instructors teaching in the individual study phases showed that for the first study phase there are significant differences between the five medical faculties (χ^2^=20,87, df=4, p=.00, w=.212): at two locations far fewer first-phase instructors participated in the survey than at other locations. For the second study phase (χ^2^=2,85, df=4, p=.58, w=.078) and the final practical year (χ^2^=6,80, df=4, p=.15, w=.121) there were no significant differences between the locations. In regard to gender, it was seen that comparatively more female instructors teaching in the first phase (χ^2^=3,98, df=1, p=.05, w=.093) and more male instructors teaching in the final year (χ^2^=21,55, df=1, p=.00, w=.216) participated in the survey. In terms of the second study phase, there are no significant differences regarding the distribution by gender (χ^2^=1,34, df=1, p=.25, w=.054). Due to data protection requirements, data was not collected on which subjects instructors taught or which teaching formats they use (e.g. lecture, seminar, etc.), since this could make it possible to determine individual identities.

#### Statistical analysis

The statistical analysis was performed using IBM SPSS, Version 24 (IBM SPSS Statistics for Windows, Version 24). To check the response distributions, individual item analysis and the chi-square test were carried out.

## Results

We present some of the main results of the survey in the following.

### Importance of feedback

A majority of instructors find feedback to be important (23%) or very important (72%). A total of 5% of the teachers do not find feedback important. No significant differences could be determined when rating the importance of feedback depending on the gender of the respondents (χ^2^=2,61, df=3, p=.46, w=.075) or depending on location (χ^2^=12,21, df=12, p=.43, w=.162).

#### Frequency of use for feedback methods

**Figure 1:**
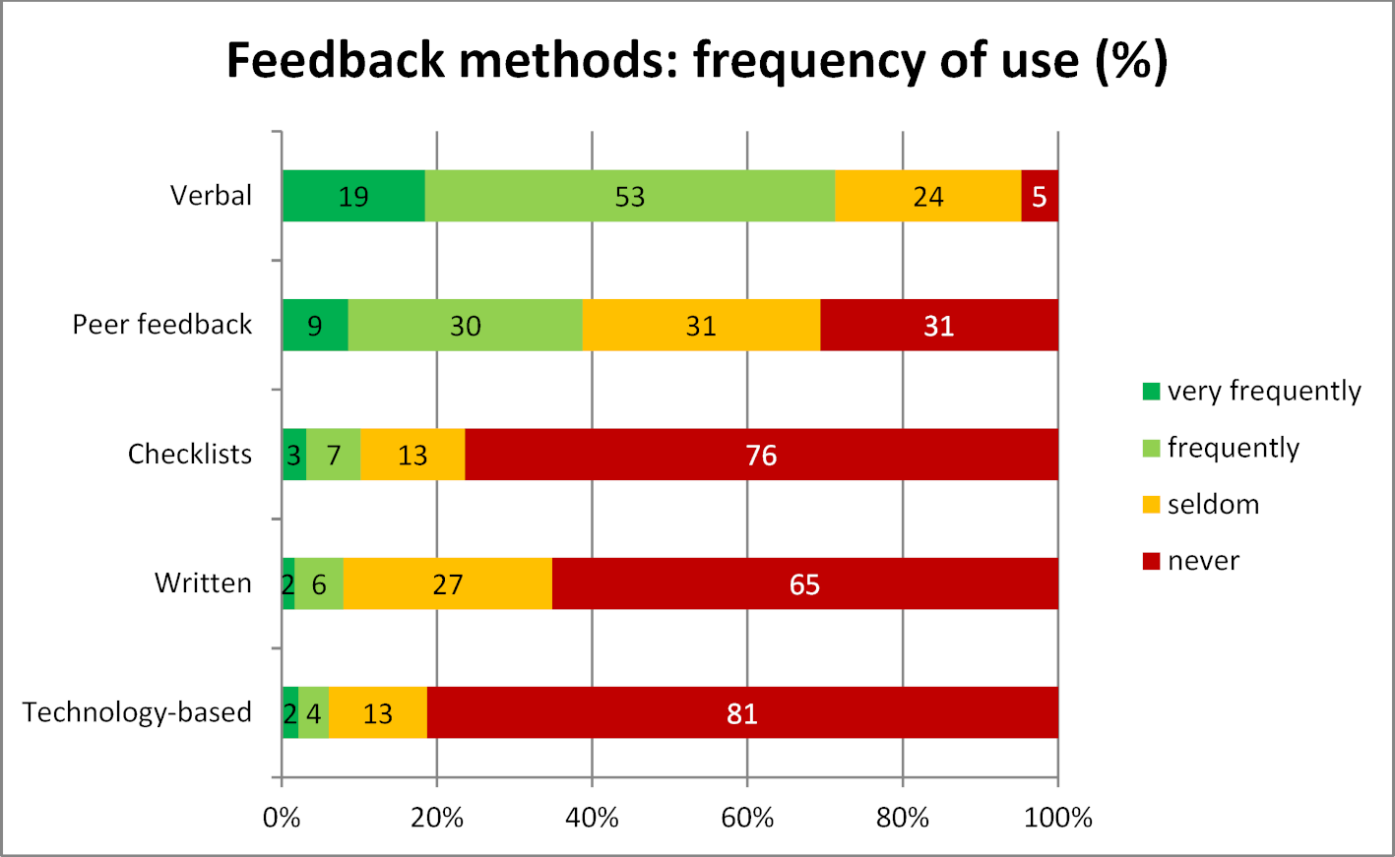
How frequently do you use the following feedback methods? (N=464)

Figure 1 (Fig. 1) shows that verbal feedback is the method most commonly employed by the instructors. Peer feedback follows in order of frequency of use. Relatively few instructors used checklists (e.g. mini-CEX, DOPS or CEC), written or technology-based feedback to inform students how well they were performing. Analysis of the extent to which the frequency of use of the feedback methods differs among the specific study phases did not yield any significant differences between the study phases.

#### Reasons for not using feedback methods

The respondents who reported never using a method were asked for the reasons why they did not use the method. One reason could be cited per method marked as never being used. The percentages for the reasons that methods were not used refer to the respondents who stated that they never used a particular method (see table 1 (Tab. 1)).

Only very few respondents indicated that they give no verbal feedback (n=22 or 5%), In other terms, most of the respondents (95%) give this type of feedback at least seldom or even more frequently. Peer feedback is not used by 31% of the instructors, of which 23% do not use this method because they are unfamiliar with it and 21% because they do not consider the method necessary for their courses. A total of 76% of the instructors reported that they did not use checklists. The reason most often cited for this is that the method is unfamiliar (56%). Other analyses show that 65% of the surveyed instructors do not use the method of written feedback. Of these, 31% consider written feedback unnecessary for their courses. Twenty-three percent do not use this method for reasons of time constraints. Of the methods covered by the survey, the most infrequently used is technology-based feedback. A total of 81% of those surveyed do not use this method. Regarding this, 24% of the instructors indicated that they were not familiar with the method and 35% identified technical reasons as the reason for not utilizing it.

#### Familiarity with feedback methods

**Figure 2:**
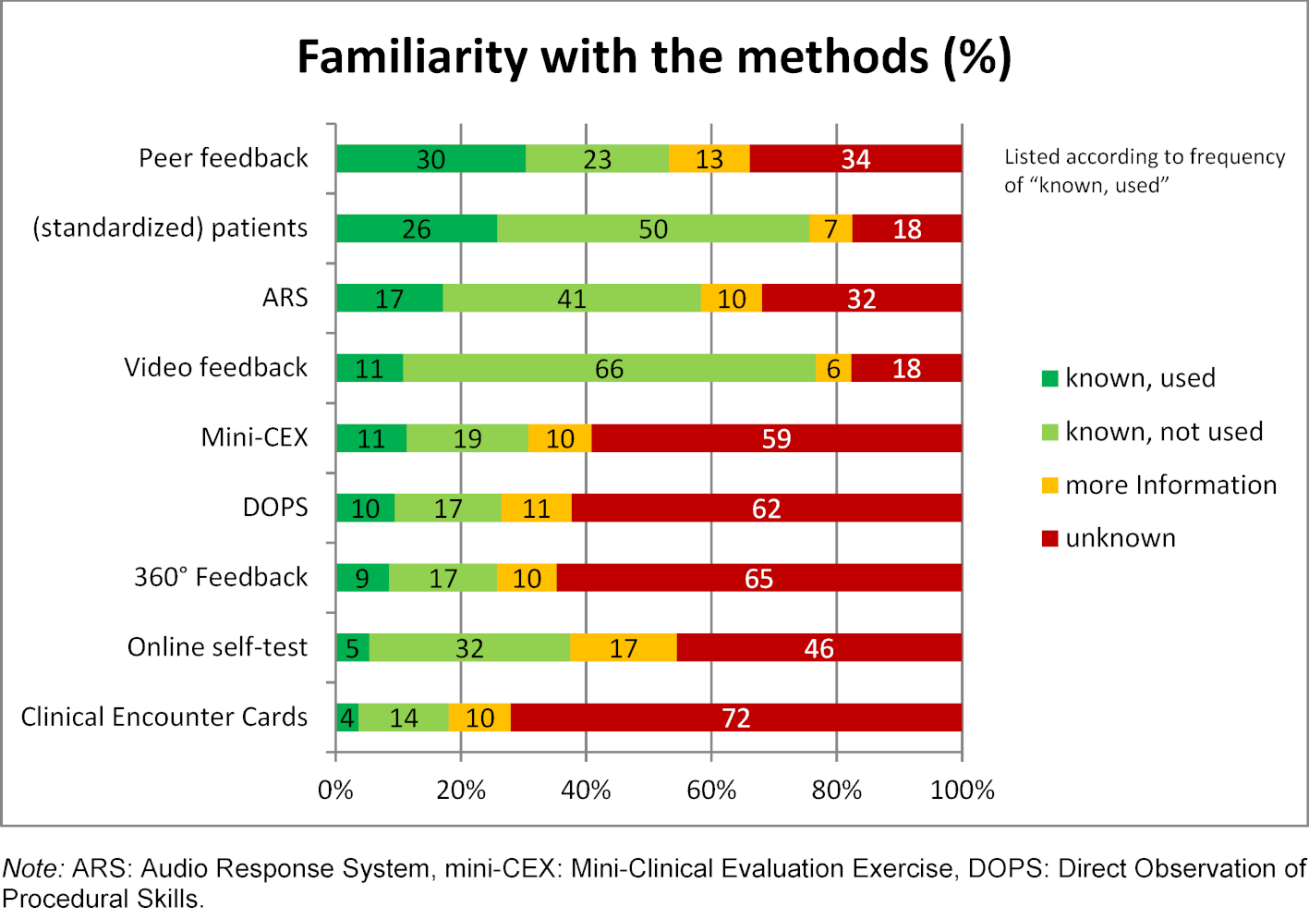
How familiar are you with the following feedback methods or instruments? (N=464)

As figure 2 (Fig. 2) shows, video feedback, feedback from (standardized) patients, audience response systems and peer feedback are the most well-known feedback methods among the instructors. In terms of familiarity with the method of providing feedback from standardized patients, there were significant differences between instructors who taught courses in the first and second study phases (χ^2^=21,82, df=6, p=.001, w=.217). Instructors who taught first-phase courses more often stated that they were unfamiliar with this method. The most frequently used methods in teaching are peer feedback, feedback from (standardized) patients and audience response systems.

Several feedback methods are not known to the majority of the instructors. 360° feedback was unknown to 65% of those surveyed; this lack of familiarity is also very relevant for the checklists. CEC is unknown to 72% of the instructors. A total of 62% are not familiar with DOPS and 59% of the lecturers are not familiar with mini-CEX.

#### Feedback situations

We also collected data on the situations in which the instructors give students feedback. By far, the instructors most often gave feedback in learning groups during course sessions (71% frequently or very frequently). Likewise, instructors relatively frequently encouraged students to give each other feedback (40%). Listed in the order of frequency of use are feedback as part of assessments or tests (38%) and feedback during breaks (34%). In contrast to this, most respondents stated never giving feedback on a learning portfolio (75%) or online via a learning platform (89%). Instructors were also asked if they gave students the option to evaluate them: 20% of instructors enable their students to give them feedback very frequently, 48% frequently, 24% seldom and 8% never.

#### Desire for more information on the topic of feedback

A total of 55% of the instructors desire more training, collaboration or information on the topic of feedback; most often mentioned as the desired form were seminars, e-learning and blended learning formats or informational materials.

## Discussion

Feedback is of great importance to competency-based teaching since students must be informed about their current level of proficiency, as well as their strengths and weaknesses, in order to further develop their competencies [5]. The results of the study show that the relevance of feedback from instructors in medical education is recognized by the majority.

Verbal feedback and peer feedback were the methods most often used by the instructors to inform students about their proficiency. In particular, the giving of verbal feedback appears to be a relatively regular component of medical education. In the case of verbal feedback it must be noted that due do its potentially casual nature it is possible that students do not perceive the information received as feedback. As a consequence, it is important that instructors clearly indicate to students when information is to be understood as feedback [10]. So that feedback can have its intended effect and meet with students’ acceptance, it is furthermore relevant that feedback from instructors is imparted in an adequate manner according to established rules for giving feedback [5], [18], [19], [20]. The same should also apply when students give instructors feedback.

At present, some feedback methods are hardly being used in medical education. While verbal feedback and peer feedback are used relatively frequently, checklists, technology-based and written feedback are not used by most of the survey respondents to give students feedback on their progress (see figure 1 (Fig. 1)). One reason for this could be that peer feedback and, in particular, verbal feedback can be given spontaneously, informally and without much preparation (even if the use of these methods should be structured and with prior preparation). The use of checklists, written and technology-based feedback, in contrast, require more advance preparation and cannot take place without prior arrangements, e.g. questions for the audience response system during lectures.

When analyzing the results of the frequency with which feedback methods are used, it must be taken into consideration that the question how frequently a method is used elicits a subjective estimation from the instructor and that the question is not asked if the particular method is currently being used. Also, it must be noted that specific learning assignments that are referenced, such as learning portfolios and learning platforms, are not being extensively used at individual medical schools currently or that the instructors are not involved in their use. This could explain why so few instructors reported using these methods to give feedback. To use these methods, a strategy for their use must be in place at the faculties, for instance in the case of digital teaching and learning.

Instructors indicated that the non-use of technology-based feedback methods is primarily due to technical reasons (see table 1 (Tab. 1)). One main reason for the high number of mentions for technical reasons, due to which the technology-based feedback is not used, could be that the technical expertise to use this method is lacking. Other explanations could be that the technical equipment needed for audience response systems is not available in every lecture hall or seminar room or that the installation and use of such systems is perceived as involving too much effort. Yet another reason could be that the method is not suitable for the course the instructor is teaching, for instance, if the course is offered in a clinical setting.

Checklists are not used particularly because many instructors are unfamiliar with this method, although checklists are relatively easy to implement and the feedback that is given using them is considered reliable if there are a certain number of cases [15], [16], [17]. For this reason, further steps must be taken to make feedback methods, their relevance and the opportunities to employ them more familiar. Instructors should be informed about the advantages, disadvantages, and potential situations in which individual methods can be used. In addition, the familiarity of the basic rules for giving feedback described in the literature should be increased [5], [18], [19], [20]. The finding that feedback for students from instructors is not yet a self-evident part of medical education corresponds with other studies, from which it can be gleaned that medical students seldom receive feedback as a result of an observed situation [12], [13].

One option for increasing the familiarity of feedback methods and rules could be printed flyers, similar to the information cards developed for the MER*LIN* project at the University of Freiburg for the mentors and students regarding the final practical year. In addition, better reference to existing or the development of new informational materials, manuals and films, etc. would be valuable. These materials could briefly describe selected feedback methods frequently found and discussed in the literature, along with their potential uses, strengths and weaknesses. Compiling a list of best-practice examples would be valuable to boost motivation and clearly present the possibilities.

In summary, the frequency with which feedback is given is not by itself of relevance, but rather the quality of the feedback is important. Also, attention should be paid that the selected feedback methods are suitable for a specific course format (e.g. audience response systems for lectures) and that the choice of feedback methods depends on the learning objectives to be met (e.g. DOPS for practical skills). To implement a constructive feedback process, it is important to realize that feedback always takes place as an interaction [5], [21]. When giving and receiving feedback, it is important that appropriate communication rules be followed [5], [22], [23].

### Study Limitations

Dissemination of the survey was achieved largely through the forwarding of emails by the teaching coordinators. An exception to this was seen at one location where all of the instructors could be contacted directly. A limitation exists in that by doing this, it is impossible to determine in the course of recruiting how many instructors in total received the survey or the link or the numbers of instructors for each location. Consequently, no statement can be made regarding the response rate or the representative nature of the study. For reasons of data protection, we did not ask instructors which subjects they taught or which teaching formats they use.

## Conclusions

The use of feedback methods in medical education is capable of being expanded and a large majority of instructors consider feedback to be important or very important in the teaching of medicine. Hence, there should not be any obstacles to a stronger use of feedback methods. So that the use of feedback methods can indeed be expanded, familiarity with the methods must be increased and the opportunities to use them must be demonstrated.

All of the medical schools in Baden-Wurttemberg are constantly working to develop targeted measures to improve the use of feedback methods. To ascertain the effectiveness of these measures, the survey will be repeated after a reasonable time in order to determine potential changes in the use of feedback methods.

## Note

The survey can be requested from the* Baden-Württemberg Competence Centre for Evaluation in Medicine, Freiburg*.

## Funding

This study received funding as part of the BMBF-sponsored MER*LIN* (Medical Education Research – *Lehrforschung im Netz BW*) project at the medical faculties in Freiburg, Heidelberg, Mannheim, Ulm und Tuebingen, with primary responsibility for the project lying with Freiburg; number: 01Pl12011A.

## Ethics

Research was carried out in compliance with the Helsinki Declaration and approved by the Ethics Commission at the Medical Faculty of the Albert-Ludwigs-Universität Freiburg (149/17). All participants were informed in writing about the study and gave their consent. Participation was voluntary. No personal data of the participants has been reported in this study.

## Acknowledgements

We wish to thank all of the people working on the MER*LIN* project for their valuable collaboration and ideas. We also extend our gratitude to all of the teaching coordinators and instructors for participating in this study.

## Competing interests

The authors declare that they have no competing interests. 

## Figures and Tables

**Table 1:**
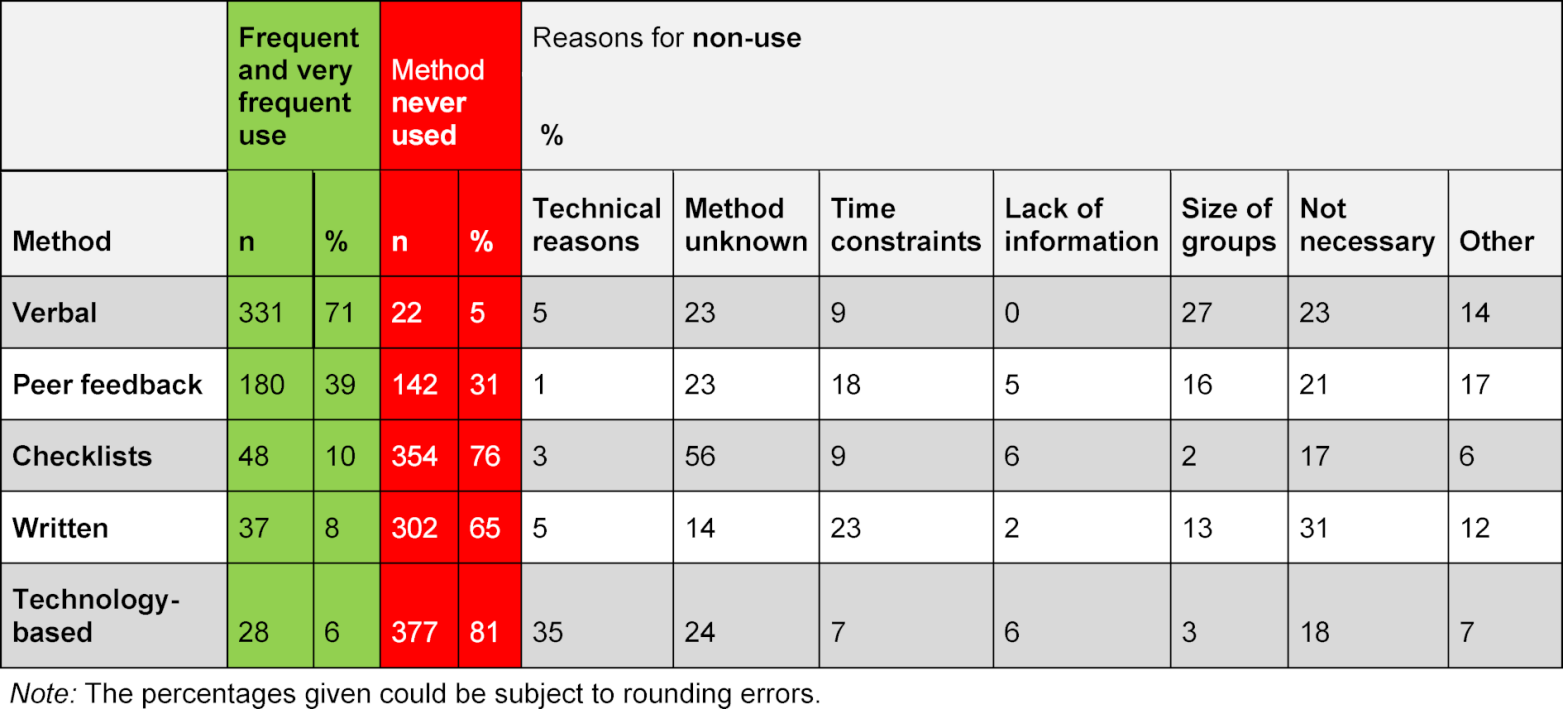
What are the main reasons why you do not use a particular method? (N=464)
